# Modeling Cell Communication in Cancer With Organoids: Making the Complex Simple

**DOI:** 10.3389/fcell.2020.00166

**Published:** 2020-03-18

**Authors:** Elena Fiorini, Lisa Veghini, Vincenzo Corbo

**Affiliations:** ^1^Department of Diagnostics and Public Health, University of Verona, Verona, Italy; ^2^Department of Medicine, University of Verona, Verona, Italy; ^3^ARC-Net Research Centre, University of Verona, Verona, Italy

**Keywords:** tumor microenvironment, co-culture systems, extracellular vesicles, organoids, extracellular matrix

## Abstract

Homotypic and heterotypic interactions between cells are of crucial importance in multicellular organisms for the maintenance of physiological functions. Accordingly, changes in cell-to-cell communication contribute significantly to tumor development. Cancer cells engage the different components of the tumor microenvironment (TME) to support malignant proliferation, escape immune control, and favor metastatic spreading. The interaction between cancerous and non-cancerous cell types within tumors occurs in many ways, including physical contact and paracrine signaling. Furthermore, local and long-range transfer of biologically active molecules (e.g., DNA, RNA, and proteins) can be mediated by small extracellular vesicles (EVs) and this has been shown to influence many aspects of tumor progression. As it stands, there is a critical need for suitable experimental systems that enable modeling the cell-to-cell communications occurring in cancer. Given their intrinsic complexity, animal models represent the ideal system to study cell-to-cell interaction between different cell types; however, they might make difficult to assess individual contribution to a given phenotype. On the other hand, simplest experimental models (i.e., *in vitro* culture systems) might be of great use when weighing individual contributions to a given phenomenon, yet it is imperative that they share a considerable number of features with human cancer. Of the many culture systems available to the scientific community, patient-derived organoids already proved to faithfully recapitulate many of the traits of patients’ disease, including genetic heterogeneity and response to therapy. The organoid technology offers several advantages over conventional monolayer cell cultures, including the preservation of the topology of cell-to-cell and cell-to-matrix interactions as observed *in vivo*. Several studies have shown that organoid cultures can be successfully used to study interaction between cancer cells and cellular components of the TME. Here, we discuss the potential of using organoids to model the interplay between cancer and non-cancer cells in order to unveil biological mechanisms involved in cancers initiation and progression, which might ultimately lead to the identification of novel intervention strategy for those diseases.

## Introduction

Homotypic and heterotypic interactions between cells are of crucial importance in multicellular organisms for the maintenance of physiological functions, including embryonic development, neurotransmission, wound healing, and inflammation. An individual cell can interact with a heterogeneous group of cells in multiple ways, that is through physical contact, ligand–receptor interactions, cellular junctions or through secreted mediators. Non-cellular components of the extracellular microenvironment add another layer of complexity to this phenomenon. Indeed, the extracellular matrix (ECM) has many functions besides providing mechanical support for organs, tissues, and individual cells. ECM, which is rich in collagens, proteoglycans, hyaluronic acid, laminins and fibronectin, is constantly remodeled in physiological processes, especially during development and wound repair, as well as in many diseases. Its degradation and reassembly regulate cell proliferation, differentiation, survival, and migration (Daley et al., 2008). ECM proteins can bind either to adhesion receptors (e.g., integrins) to transduce intracellular signals or growth factors to regulate their presentation to cells (Hynes, 2009). The cell communication process and its regulation acquire major importance in the cancer contest, where cancer cells interact and communicate with a variety of non-cancerous cells, including normal epithelial cells, immune cells, endothelial cells and fibroblasts, which collectively constitute the tumor microenvironment (TME). In addition, recent evidences suggest a major role for a cell-to-cell communication system that involve extracellular vesicles (EVs) transferring biologically active molecules, which can substantially modify the cellular behavior of recipient cells (Ratajczak et al., 2006; Valadi et al., 2007). As it stands, complex interactions that occur in the TME need to be taken into account when modeling cancer. Driven by recent advances in cell culture technology and EVs isolation methods, we review how next generation culture systems, in particular organoids, represent a promising tool to help modeling cell-to-cell communication. Finally, we highlight some of the challenges that researchers are still facing with organoid cultures, including lack of standardization of ECM and media being used to support organoid formation and expansion.

## Models to Study Cancer Cells Interactions Within the TME

Animal models represent the gold standard to study interactions between cancer cells and the microenvironment. Transplantation of patient-derived tumor’ tissue or cells in immunocompromised mice (Patient-derived xenografts, PDXs) is widely used in preclinical studies to model drug responses. Even if PDXs are considered a good avatar of primary human tumors, recent evidences suggest that generation of xenografts is associated to selection of genetic clones (Eirew et al., 2015; Ben-David et al., 2017). Furthermore, PDXs fail to properly recapitulate the interaction with the human immune system due to species-specific differences. Genetically engineered mouse models (GEMMs) spontaneously develop tumors in an immune-proficient microenvironment, yet their fast progression and homogeneous genetic background fail to replicate properly the heterogeneity of human diseases (Cheon and Orsulic, 2011). Humanized immune reconstituted (HIR) mice offer the possibility of transplanting patients’ tumors in a host that at least partially recapitulate the human immune system, thus providing a more realistic representation of the disease. Nonetheless, the use of HIR mice models is less straightforward, due to species-specific differences and incorrect representation of the patients’ immune system (Rangarajan and Weinberg, 2003).

Although animal models provide a more realistic platform to model the disease as a whole, their intrinsic complexity makes difficult assessing individual contributions to a given phenotype. On the other hand, *in vitro* two-dimensional (2D) and three-dimensional (3D) cell culture systems represent a facile platform to understand causative relationships in cancer through different type of perturbation analyses (i.e., genetic and non-genetic).

Conventional monolayer cell culture systems have been of tremendous importance for the current understanding of many diseases, including cancers; however, they suffer from several limitations making them inappropriate to correctly model patients’ tumors. A comprehensive evaluation of the advantages of 3D culture systems over 2D systems is beyond the scope of this review, and it has been extensively described elsewhere (Fong et al., 2016; Avnet et al., 2019; D’Agosto et al., 2019; Yang et al., 2019).

The most commonly used 3D culture models are spheroids and organoids. Spheroids are cell aggregates or spheres cultured primarily in suspension, which are likely enriched for stem-like cell population (Weiswald et al., 2015). This technology can be applied to both cancer cell lines and patient-derived tumor cells, but it is not applicable to normal cells from many tissues. The use of spheroids ranges from drug screening to modeling immune interactions (Katt et al., 2016). Spheroids partially compensate deficiencies of monolayer cultures; the formation of an aggregate creates a gradient of nutrients, oxygen and metabolites, and models cell-to-cell and cell-to-matrix interactions (Costa et al., 2016). Beyond a few exceptions (i.e., secretory acini spheroids) (Wu et al., 2011), the random aggregations of cells, and the consequent lack of organization in tissue-like structures, makes spheroids poor models of epithelial tissues (Torras et al., 2018). On the other hand, we defined organoids as 3D cultures derived directly from the dissociation of specialized epithelial tissues, from embryonic stem cells (ESCs) or induced pluripotent stem cells (iPSCs), all showing self-renewal and self-organization capabilities (Lancaster and Knoblich, 2014). Furthermore, organoids are capable of preserving many relevant features of *in vivo* tissue physiology. Studies of tissue morphogenesis and early 3D cultures of mouse mammary primary cells (Moscona and Moscona, 1952; Bissell, 1981) set the ground for the subsequent development of the organoid technology. The laboratory of Mina Blissell was the first to show that primary epithelial cells from mouse mammary glands could self-organize in glandular structures and express milk proteins when cultured in a basement membrane matrix (BM). The BM used in that seminal work was isolated from mouse Engelbreth-Holm-Swarm (EHS) sarcoma (Barcellos-Hoff et al., 1989), currently branded as Matrigel^®^ (Swarm, 1963; Orkin et al., 1977), and mainly composed by a mixture of collagen type IV, laminin, heparan sulfate proteoglycans, and entactin.

Few years later, the same method was applied to the propagation of human cells derived from both normal and tumoral tissues (Petersen et al., 1992). Notwithstanding the important earlier studies, the first organotypic models were reported in 2008 and in 2009 to enable growing cortical neurons (Eiraku et al., 2008) and intestinal epithelium (Sato et al., 2009), respectively. In 2009, the Clevers’ group (Sato et al., 2009) described the method for the generation of organoid cultures from individual Lgr5+ stem cells isolated from mouse intestinal tissue. The Lgr5+ cells are embedded in Matrigel^®^ and overlaid with a defined culture medium supplemented with several growth factors and morphogens that substitute for stromal cues. The resulting organoids consisted of a monolayer of epithelial cells surrounding a central lumen as well as of protrusions containing stem cells and differentiated Paneth cells that form the stem cells niche. Hereafter, the procedure was adapted for the generation of organoids from human small intestine (Sato et al., 2011), which showed some differences compared to mouse organoids: human organoids exhibited a more cyst-like morphology with a concomitant increased need for Wnt signaling stimulation. The establishment of organoid cultures have been later reported from several human normal epithelial tissues (Barker et al., 2010; Sato et al., 2011; Huch et al., 2013a, b; Lancaster et al., 2013; Pringle et al., 2016). The possibility of growing normal epithelial cells without the need for genetic immortalization (e.g., through exogenous expression of viral genes) probably represents one of the major advantages of the organoid technology. Normal tissue-derived organoids might be used to study the mutual influence between healthy and cancer cells and to discover cancer-specific interactions within the TME. Nonetheless, they offer new opportunities for regenerative medicine applications (Grassi et al., 2019). Building on the seminal studies of the Clevers’ group, modifications of the original procedure were used for culturing human organoids derived from hPSCs (Spence et al., 2011) as well as from neoplastic tissues. To date, patient-derived organoids have been established from different cancer tissues, including colon (Sato et al., 2011), brain (Linkous et al., 2019), gastric (Wang K. et al., 2014), pancreatic (Boj et al., 2015), prostate (Drost et al., 2016), liver (Broutier et al., 2017), breast (Sachs et al., 2018), esophageal (Li et al., 2018), kidney (Schutgens et al., 2019), and lung (Sachs et al., 2019) cancers. Another organoid culture system has been reported by the Calvin Kuo’s lab, known as air liquid interface (ALI) system (Ootani et al., 2009). In this system, organoids are grown in a stromal matrix constituted by type I collagen that has an apical side, exposed to the air, and a basolateral side in contact with the media (Ootani et al., 2009). Although they first applied it to mouse-derived normal intestinal epithelia, the same method was then applied to normal and tumoral tissues derived from both mouse and human (Li et al., 2014; Usui et al., 2016, 2018; Yokobori et al., 2016; Giandomenico et al., 2019).

## Tumor Organoid Co-Cultures Model the Interactions With TME

**FIGURE 1:**
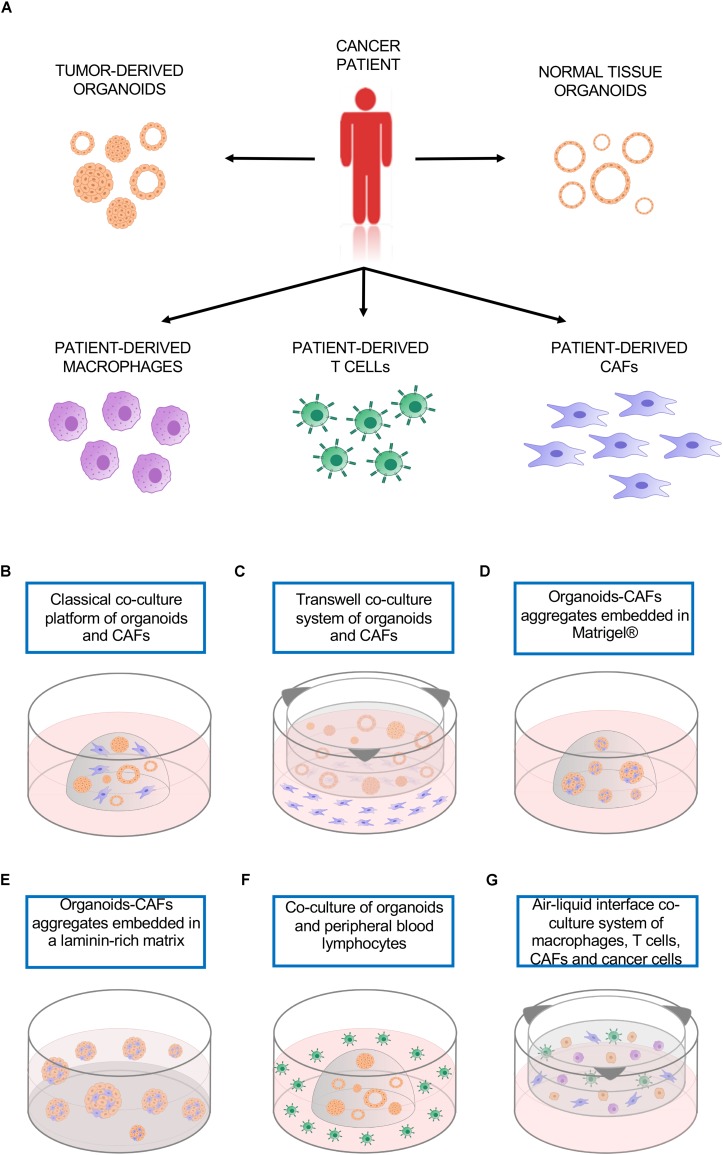
Co-culture platforms. **(A)** Tumor organoids as well as cultures of different non-neoplastic cell types can be established from the same patient and used to model cell-to-cell interactions. **(B)** Classical co-culture platform in which organoids and CAFs are embedded in the same drop of Matrigel^®^. **(C)** Transwell co-culture system in which organoids are embedded in a matrix on top of a transwell insert, CAFs are plated as monolayer culture. **(D)** Organoids-CAFs aggregates embedded in a drop of Matrigel^®^. **(E)** Organoids-CAFs aggregates embedded in a laminin-rich matrix on a Matrigel^®^ precoated well. **(F)** Co-culture of organoids and peripheral blood lymphocytes, the organoids are embedded in a drop of Geltrex^®^ while the lymphocytes are in suspension in the culture media. **(G)** Air-liquid interface co-culture system, patient-derived macrophages, T cells, CAFs, and cancer cells are embedded in collagen type I on top of a permeable insert precoated with collagen matrix. CAFs, cancer-associated fibroblasts.

Organotypic cultures, like 2D cell lines, usually contain only one cell type representing the neoplastic epithelium, therefore lacking the multi-cellular representation of the TME. This limitation can be partly solved by the ability of patient-derived cancer organoids to be easily co-cultured with a variety of cell types, including patient-derived immune cells or cancer-associated fibroblast (CAFs) (Figure 1A; Lee et al., 2007; Marusyk et al., 2016; Ohlund et al., 2017; Dijkstra et al., 2018; Neal et al., 2018; Seino et al., 2018). 3D co-culture system offers a promising tool to model the interactions between tumor cells and the other cells that compose the TME.

### Co-cultures With CAFs

Cancer-associated fibroblast constitute the major cellular component of the TME in many tumors (LeBleu and Kalluri, 2018) and can be recruited at the tumor site or differentiated from resident mesenchymal cells. Their increased expression/secretion of tumor-promoting factors, collagen, and other ECM proteins distinguish them from the normal fibroblast population. CAFs influence tumor growth and metastasis by several mechanisms. Via matrix synthesis and remodeling, they can modulate ECM stiffness and hypoxia facilitating tumor cells mobility and invasion. Through secreted molecules, such as growth factors, cytokines, chemokines and microRNAs (miRNAs), CAFs support tumor growth and induce resistance to therapy (Karagiannis et al., 2010; Zhang et al., 2018). Moreover, a direct cell-to-cell contact between CAFs and cancer cells, mediated by N-cadherin/E-cadherin adhesion complex, has been recently proved to promote tumor invasion (Labernadie et al., 2017). However, CAFs targeting experiments evidenced a more complex response than expected. For instance, the ablation of contractile isoform of alpha smooth muscle actin (α-SMA) cells in a mouse model of pancreatic ductal adenocarcinoma (PDAC) was found to reduce cancer desmoplasia, yet resulted in more aggressive and highly undifferentiated tumors, suggesting that the presence of this cell type influence the trajectory of tumor development and progression (Ozdemir et al., 2014). Similar results were obtained with the deletion of Sonic Hedgehog I, a ligand that enhances the formation of fibroblast-rich desmoplastic stroma in PDAC (Rhim et al., 2014). The contradictory results evidence the need to investigate more in deep the different subgroups of CAFs and their functions.

Pancreatic cancer organoids exemplify a culture system that can be used to model interaction between cancer cells and CAFs. Ohlund et al. (2017) developed a co-culture system that is able to recapitulate some of the features of the desmoplastic reaction as observed in patients. They demonstrated the existence of two CAFs subpopulations, which are differentially located in tumor tissues and display distinct phenotypes. Those populations were defined as myofibroblasts (myCAFs), proximal to the neoplastic cells and characterized by high levels α-SMA, and as pro-inflammatory CAFs (iCAFs), which are distally located from the cancer cells and have low expression of α-SMA and high expression of IL-6. Using two different types of co-culture, they demonstrated the possibility of modeling CAFs subtypes using an *in vitro* system. The co-culture was established using pancreatic stellate cells, which are resident mesenchymal cells expressing fibroblast-activation protein α (FAP), and pancreatic organoids established either from patients or mouse tumors. When both cancer cells and fibroblasts are embedded together in Matrigel^®^ (Figure 1B), the close contact induced an activation of quiescent PSCs, with the acquisition of myCAFs phenotype. When the two cell types are physically separated (Figure 1C), yet sharing the growth medium, there was emergence of the iCAFs phenotype. The results obtained with the two co-culture methods sustained the concept of CAFs intratumoral heterogeneity, further supported by recent single cell RNA sequencing data on bulk PDAC tissue, that identified a third subtype, named antigen presenting CAFs (apCAFs), characterized by the expression of MHC class II related genes. Interestingly, all these subtypes are dynamic and reversible (Elyada et al., 2019).

An additional type of co-culture has been employed to investigate the role of CAFs in supporting the growth of pancreatic cancer organoids (Seino et al., 2018). In this study, pancreatic organoids and CAFs were dissociated into single cells and then aggregated before being transferred in a Matrigel^®^/collagen mixture (Figure 1D). This physical attachment allowed the CAFs to supply the organoids with the niche factor necessary for their growth. In agreement with these findings, a 3D co-culture model of prostate organoids and stroma enhanced the formation and induced a change in the morphology of organoids from spherical shape in mono-culture to branched acini structure in co-culture, more similar to what is observed *in vivo* (Richards et al., 2019).

Cancer-associated fibroblast represent an abundant non-neoplastic cell type also in breast cancers, in which they are implicated in tumor progression and resistance to both chemo- and targeted-therapies (Paraiso and Smalley, 2013). A 3D co-culture approach has been employed to investigate the modifications in gene expression and cell metabolism of breast cancer cells induced by the interaction with CAFs. After pre-coating the plate with a thin layer of Matrigel^®^, the two cell types were aggregated and cultured on top of the laminin-rich ECM (Lee et al., 2007; Figure 1E). This method uncovered the effects of CAFs in attenuating the accumulation in epithelial cells of Lapatinib, a tyrosine kinase inhibitor used in the treatment of breast cancer (Marusyk et al., 2016).

### Co-cultures With Immune Cells

Immune cells have antithetic functions in the TME. Innate and adaptive immune systems both exert their influence on TME and in turn are affected by TME. Macrophages, one of the most abundant cell types in the tumor stroma, are innate immune system phagocytic cells that can have pro- or anti-tumor activity. M1 macrophages are pro-inflammatory and considered tumor suppressive, but when they interact with the TME they undergo a phenotypic switch toward the M2 phenotype, known to be anti-inflammatory and tumor promoting. The integration of peripheral blood-derived macrophages in a 3D co-culture system of squamous cell carcinoma allowed the modulation of the macrophages phenotype. The classical protocol for polarization of macrophages involve the use of recombinant INF-γ and LPS to induce the M1 phenotype, while IL-4 stimulation promotes the M2 phenotype. Interestingly, the prolonged co-culture with cancer cells was sufficient to cause the spontaneous switch of macrophages phenotype toward M2, even in absence of IL-4 (Linde et al., 2012). Adaptive immune cells, like CD8+ T cells, are subjected to TME influence as well. CD8+ T cells are major actors of anti-cancer immunity; their infiltration in TME has been reported to correlate with better prognosis in several malignancies such as melanoma, ovarian, pancreatic, breast and colorectal cancers (Fukunaga et al., 2004; Sato et al., 2005; Neagu, 2012; Ziai et al., 2018). CD8+ T cells act by recognizing tumor-associated antigens presented to their T cell receptor by major histocompatibility complex (MHC). However, tumors often activate immune evasion mechanisms that include secretion of immunosuppressive cytokines, MHC downregulation or resistance to T cell-mediated cell death (Khong and Restifo, 2002). The programmed death 1 (PD-1) immune checkpoint is a key player in the tumor-mediated immune evasion mechanisms. When PD-1 interacts with one of its ligands, PD-L1 or PD-L2, activates the recruitment of SHP-2, which functions suppressing T-cell receptor signaling, inducing tumor-specific T-cells exhaustion or apoptosis. In tumor, PD-L1 is expressed not only by cancer cells but also by cells of TME or antigen presenting cells. PD-1/PD-L1 complex is one of the targets of cancer immunotherapy based on immune checkpoint inhibitors (ICI), which aims at unleashing anti-tumor responses by activating immune cells. Unfortunately, only subsets of epithelial cancers patients have shown benefit from cancer immunotherapies based on ICI. While genome sequencing data have largely contributed to the identification of biomarkers predictor of response, the molecular mechanisms of sensitivity/resistance are still unclear. Recently, Dijkstra et al. (2018) described a patient personalized *in vitro* model that enable the induction and the analysis of tumor specific T cell responses. Organoids were established from mismatch repair deficient colorectal cancer and from non-small-cell lung cancer patients, while T lymphocytes were isolated from patient’ peripheral blood. The co-culture of tumor organoids and peripheral blood lymphocytes (Figure 1F) allowed the expansion of tumor-reactive T cells, which were able to kill tumor organoids but not the ones derived from healthy tissue. This platform permits the study of tumor and T cells interactions with the potential to uncover the determinants of sensitivity or resistance to immunotherapy in a personalized manner. Another platform has been described by Jenkins et al. (2018) and is based on a 3D microfluidic device, which enabled preservation of both tumor infiltrating lymphoid and myeloid cells along with tumor-spheroids. Using this system, patient-derived organotypic tumor spheroids proved to be a good model to study tumor-immune interactions and responses to PD-1 blockade. In addition, a peculiar co-culture method based on ALI system (Neal et al., 2018) permitted the propagation of patient-derived organoids and tumor-infiltrating lymphocytes. In this system, minced tumor tissues are resuspended in type I collagen and plated on a permeable insert precoated with collagen matrix (Figure 1G). The ALI culture system was found to preserve the native stromal populations, including fibroblasts, macrophages and lymphocytes, for up to 2 months. Importantly, tumor-infiltrating lymphocytes maintained the T cell receptor repertoire observed in patients and the patient-derived organoids response to anti-PD-1 Nivolumab *in vitro* was comparable to what has been observed in clinical trials.

## New Horizons of Organoids Application: Modeling EVs Release

Communication between cancerous cells and TME is also achieved through release of extracellular vescicles (EVs), a heterogeneous population of double-layer phospholipid membrane vesicles released by most cell types. The different populations of EVs can be discriminated by vesicle size, biogenesis, and release pathway. Microvesicles are vesicles with a diameter ranging from 200 to 2000 nm that originate directly from the budding of the cellular membrane (Li et al., 2012). Exosomes are smaller vesicles, with a diameter less than 200 nm, derived from intracellular endosomal compartment, called multivesicular body, and released by the fusion of the multicellular body with the plasma membrane (Kowal et al., 2014). Another type of EVs is represented by apoptotic bodies that originate during the late stages of programmed cell death and containing part of cytoplasm (Turiak et al., 2011). Several findings support the hypothesis that the bioactive molecules encapsulated in the EVs are capable of modifying the functions of the recipient cells. Tumor-derived EVs, also referred as *oncosomes* due to the fact that their cargo is composed by oncogenic macromolecules (Meehan et al., 2016), have been proven to induce changes in the phenotype of neighboring as well as distant cells, promoting tumor growth and local invasion in different cancer types (Webber et al., 2015; Fang et al., 2018; Naito et al., 2019). In addition, atypically large (1–10 μm in diameter) EVs derived from amoeboid tumor cells have been recently identified in the circulation of different mouse models of prostate cancer as well as in the biological fluids of metastatic prostate cancer patients (Di Vizio et al., 2012). This so-called large oncosomes (LOs) are not detected in samples from healthy donors and appear to originate from the shedding of non-apoptotic plasma membrane blebs (Minciacchi et al., 2015, 2017).

Extracellular vescicles are also released by stromal cells in the TME to exert their influence on cancer cells. ANXA6+ (Annexin 6/LDL receptor-related protein-1/thrombospondin-1 complex) CAFs-secreted EVs enhanced tumor cell aggressiveness in pancreatic cancer (Leca et al., 2016). Fibroblast-derived exosomes induced mobilization, invasion (Luga et al., 2012), resistance to therapy and tumor recurrence in breast cancer (Boelens et al., 2014), while CAFs EVs-mediated transfer of proteins and lipids enhanced cancer proliferation rate of human prostate cancer and melanoma cell lines (Santi et al., 2015). The EVs transfer of surface receptors or intracellular material has been also associated with immunosuppressive and tumor-promoting functions. The expression of immunomodulatory molecules on exosomes surface contributes to tumor immunosuppression. Fas ligand on exosomes induces T-cell apoptosis in a prostate cancer cell model (Abusamra et al., 2005), while expression of PD-L1 suppresses the function of CD8+ T cells thereby enhancing tumor growth in a xenograft mouse model of melanoma (Chen et al., 2018). Moreover, melanoma EVs interact with B lymphocytes in lymph nodes and mediate alteration of antitumor immunity promoting cancer progression (Pucci et al., 2016). Additional evidences proved that lung cancer-derived exosomes engineered to overexpressed CD40 ligand, part of a critical signaling for the activation of dendritic cells, induced dendritic cells maturation with a consequent increase in T cells proliferation and antitumor activity (Wang J. et al., 2014).

Based on the “Seed and Soil” hypothesis proposed by Paget in 1889, several works have been published demonstrating that tissues distant from the site of the primary tumor, undergo processes of adaptation to become permissive for the growth of cancer cells. The Lyden’ s group unveiled the role of exosomes in this process (Peinado et al., 2012; Costa-Silva et al., 2015; Hoshino et al., 2015). They proved that the transfer of MET oncoprotein by melanoma-derived exosomes is able to educate bone marrow progenitor cells to become pro-metastatic, leading to the discovery of an exosome signature with prognostic and therapeutic potential (Peinado et al., 2012). More recently, they described the sequential steps necessary for the formation of the pre-metastatic niche in pancreatic cancer, uncovering MIF as potential prognostic marker for liver metastasis (Costa-Silva et al., 2015). Moreover, proteomic analysis of tumor-derived exosomes revealed how the exosomal integrin signature is determinant for the formation of the pre-metastatic niche in specific tissues, explaining why tumors may have distinct metastatic tropisms (Hoshino et al., 2015). Tumor-derived EVs are also implicated in the remodeling of the ECM. EVs isolated from ovarian cancer cell lines were able to compromise the mesothelium integrity and to promote peritoneal dissemination of tumor cells in mice (Yokoi et al., 2017).

**FIGURE 2:**
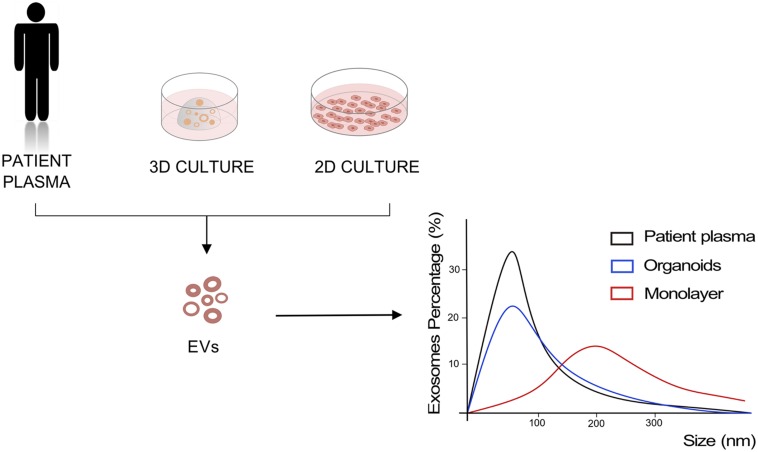
Models to study EVs. Representation of EVs size analysis for 2D and 3D culture of cancer cells compared to EVs derived from patient plasma.

Given increasing evidences of a critical role of EVs in driving cancer progression, implementation and refinement of *in vitro* system to model EV-mediated cell communication is urgently needed. Monolayer cell cultures have been historically used to investigate the content and role of EVs in cancer, and most of data reported above have been generated using 2D cell culture systems. Rocha et al. (2019) have recently compared biochemical features, small RNA transcriptome, and proteome of EVs released by two gastric cell lines when cultured in 2D and 3D conditions to uncover the effects of the culture system on EVs. They observed that 3D culture conditions affect the size of the released EVs, inducing the production of smaller vesicles, with size and concentrations comparable to the exosomes isolated from patient plasma. In addition, Ewing’s sarcoma type 1 EVs isolated from 3D cultures proved to have higher similarity to the EVs isolated from patient plasma compared to the same cancer cells cultured as monolayer (Villasante et al., 2016; Figure 2). A recent study demonstrated the ability of organoids to intake and respond to EVs (Ke et al., 2017). They showed how human normal gastric organoids responded to tumor-derived EVs content by acquiring a neoplastic phenotype. EVs-treated organoids showed increased proliferation and smaller lumens with multilayered morphology that is typical of neoplastic organoids, along with decreased expression of the tumor suppressors PTEN and AIFM3. In a colorectal cancer organotypic model, EVs purified from human colon fibroblasts grown in hypoxic conditions were able to induce increase in number of neoplastic organoids, suggesting a role of fibroblast-derived EVs in tumorigenesis (Szvicsek et al., 2019). In the same work, the introduction of *Apc* mutation into mouse small intestinal organoids by CRISPR-Cas9, and the consequent activation of Wnt pathway, enhanced EVs secretion already at the adenoma stage of the intestinal tumorigenesis (Szvicsek et al., 2019).

Another crucial advantage of 3D culture system is the possibility of modeling cell-to-matrix interactions, which appear to influence release of EVs. Indeed, it has been shown that matrix supporting organoids growth influences the production of EVs, specifically the enrichment of collagen type I increased the release of EVs in the culture media by colorectal cancer-derived organoids (Szvicsek et al., 2019).

Additionally, tumor-derived EVs presence in body fluids makes them a promising source of cancer biomarkers. EVs miRNA signatures already proved to have prognostic value in several cancer types, including colorectal cancer (Fu et al., 2018), pancreatic cancer (Goto et al., 2018), prostate cancer (Valentino et al., 2017), non-small cell lung cancers (Liu et al., 2017), and glioblastoma (Skog et al., 2008). A novel approach using organoid media supernatant has been proposed for the identification of EVs proteins present exclusively in the blood of PDAC patients, and not in patients disease-free or with benign conditions (Huang et al., 2019). The subsequent validation of EV proteins obtained from media supernatant in clinical samples demonstrated the ability of the organoid platform to enable the identification of cancer biomarkers for PDAC patients. Moreover, recent evidences suggest EVs potential role in early disease detection (Melo et al., 2015) and prediction of response to treatment (Ciravolo et al., 2012; Wei et al., 2014; Rodrigues-Junior et al., 2019). The ability to enter other cells and deliver a functional cargo, even at distant sites, without inducing toxicity makes them good therapeutic agents vehicles (Ferguson and Nguyen, 2016). EVs can be engineered to target specific cell types, deliver chemotherapeutic agents (Kim et al., 2016) and enhance antitumor immune response (Besse et al., 2016). In conclusion, although patient-derived organoids might be a promising system to study cell-to-cell interactions, additional efforts from the scientific community are expected to finally assess their potential to model EV-based communication in cancers. Given the heterogeneity of matrices used in 3D culture systems, greater efforts are also expected to analyze the different effects of matrices on the migration and biological effect of EVs.

## Organotypic System Challenges

Organoids are the *in vitro* system with a higher degree of similarity with the tissue of origin as compared to conventional monolayer cultures. Integration of different cell types through co-culture systems may help modeling the cell-to-cell interactions observed *in vivo*. However, beyond the evident advantages offered by the system, some challenges still need to be solved.

Epithelial organoid cultures usually need to be grown in a matrix that allows the formation of 3D structures and sustains their proliferation. The most commonly used matrices are biologically derived materials such as rat tail collagen I and Matrigel^®^ (Table 1). Animal-derived matrices suffer from lot-to-lot variability, both in terms of composition and structure, and are unsuitable for clinical applications (Vukicevic et al., 1992; Hughes et al., 2010). Recently, efforts have been made toward establishing synthetic ECM analogs able to replace animal-derived matrices, with the aim to obtain more characterized and less variable matrix composition, with translational potential. Synthetically produced matrices can be composed by natural or non-natural polymers. An example of natural polymers matrix is the hyaluronic acid (HA) hydrogel, a highly biocompatible material that guarantee batch-to-batch consistency and reproducible results (Burdick and Prestwich, 2011). On the other hand, non-natural polymers like polyethylene glycol (PEG) are less expensive, highly reproducible, but lack biological features necessary to reproduce natural ECM, to overcome this issue and to improve functionality, natural peptides need to be crosslinked to the scaffold (Worthington et al., 2015). Gjorevski et al. (2016) developed a synthetic hydrogel for intestinal stem cells organoids generation and growth. PEG hydrogel supplemented with an RGD (Arg-Gly-Asp) peptide supported the expansion of intestinal stem cells, preserving the stemness partly lost during Matrigel^®^ culture. Moreover, they proved that matrix stiffness and composition influence the formation and the differentiation of organoids. For this reason, they proposed the use of a mechanically static PEG to grow intestinal stem cells as spheroids, that successively need to be partially replaced by more degradable PEG to obtain the softness required for the cell’s differentiation and the formation of 3D structures. A different synthetic matrix, composed by PEG with maleimide groups at each terminus (PEG-4MAL), has been also applied for the growth, expansion and differentiation of human intestinal organoids (Cruz-Acuna et al., 2017). An initial 2D culture on Matrigel^®^-coated substrate was necessary before the encapsulation of cells in PEG-4MAL. The synthetic matrix has been engineered to obtain the optimal polymer density to support the generation of organoids derived from both ESCs and iPSCs. Furthermore, the delivery of human intestinal organoids resuspended in PEG-4MAL hydrogel into injured intestinal mucosa, improved wound repair in a mouse model, suggesting hydrogel as potential vehicle for clinical applications. To overcome the limitations of PEG hydrogel, a fibrin/laminin hydrogel has been developed for the generation and the expansion of epithelial organoids (Broguiere et al., 2018). Fibrin/laminin hydrogel proved to support long-term expansion of organoids established from different tissues, maintaining the same architecture observed in Matrigel^®^, giving proof to be a valid candidate for Matrigel^®^ replacement.

**TABLE 1:** Most commonly used matrices for the growth of 3D cultures.

**Matrix**	**Composition**	**Derivation**	**Applications**
Matrigel^®^	collagen type IV, laminin, heparan sulfate proteoglycans, entactin/nidogen, and a number of growth factors	murine Englebreth-Holm-Swarm sarcoma tumors	3D cell culture
Collagen type I	collagen type I	rat tail	3D cell culture
HA hydrogel	hyaluronic acid	synthetic	3D cell culture; clinical applications
PEG hydrogel	polyethylene glycol (PEG) hydrogel supplemented with an RGD (Arg-Gly-Asp) peptide	synthetic	3D cell culture; clinical applications
PEG-4MAL hydrogel	PEG with maleimide groups at each terminus	synthetic	3D cell culture; clinical applications
Fibrin/laminin hydrogel	fibrin, laminin	synthetic	3D cell culture; clinical applications

The other critical aspect of the organoids system is the formulation of an optimal culture media that contains the niche factors necessary for the establishment and the long-term culture of organoids. Wnt3a and R-spondin1 have been proven to be essential niche components for the establishment of patient-derived organoids from different tissues, like pancreas, liver, stomach and intestine (Clevers and Nusse, 2012). Although in the first organoid protocol it was reported the use of purified proteins (Sato et al., 2009), currently, the production of these two factors in many laboratories relies on eukaryotic cell culture engineered to secrete Wnt3a and R-spondin into their culture media due to economical constraints. The obtained conditioned media are then used for the production of the organoid culture media. Clearly, the use of conditioned media introduces elements of variability in the culture system and makes difficult comparing data generated from different laboratories. Moreover, the presence of other macromolecules, apart from the protein of interest, and batch-to-batch variability of protein activity may affect the organoid growth. Replacing conditioned media with recombinant proteins displaying similar activity may lead to the definition of standardized media conditions for the establishment of patient-derived organoids. Efforts have been recently made in this direction with the production of recombinant R-spondin (Urbischek et al., 2019) and Wnt surrogate (Janda et al., 2017).

## Concluding Remarks

The advent of 3D culture systems is enabling the systematic generation of *in vitro* models from a variety of cancers and normal tissues. Increasing evidences suggest that those cultures are genetically stable and faithfully recapitulate major molecular features of parental tissues. The integration of these new systems with improved methods for isolation and characterization of EVs offers now the opportunity of exploring the role of unconventional ways of communications between cells, which has been shown to influence many aspects of cancer biology, including metastasis. Ultimately, the possibility to isolate and culture both tumoral and non-tumoral cells from the same patient permits the assessment of malignant specific features that has never been possible before using *in vitro* system for many cancer types.

## Author Contributions

EF wrote the manuscript and performed literature search. LV prepared the figures. EF and LV drafted the manuscript. VC devised the idea and finalized the manuscript.

## Conflict of Interest

The authors declare that the research was conducted in the absence of any commercial or financial relationships that could be construed as a potential conflict of interest.
